# Hypokalemic Periodic Paralysis: A Case Report

**DOI:** 10.7759/cureus.94524

**Published:** 2025-10-14

**Authors:** Mohsin Wani, Zara Marchant, Umed Nadir

**Affiliations:** 1 Medicine, Royal Hampshire County Hospital, Hampshire Hospitals NHS Foundation Trust, Winchester, GBR; 2 Medicine, Hampshire Hospitals NHS Foundation Trust, Winchester, GBR; 3 Internal Medicine, Royal Hampshire County Hospital, Hampshire Hospitals NHS Foundation Trust, Winchester, GBR; 4 Internal Medicine, Hampshire Hospitals NHS Foundation Trust, Winchester, GBR

**Keywords:** acute muscle weakness, genetic channelopathies, genetic diagnosis, hypokalaemic periodic paralysis, hypokalemia, ion channelopathy, paralysis, potassium replacement therapy

## Abstract

Hypokalaemic periodic paralysis (HPP) is a rare neuromuscular disorder characterized by recurrent episodes of transient muscle weakness associated with hypokalaemia. The condition typically presents in childhood or adolescence.

In this case, a 17-year-old male presented with acute-onset limb weakness upon waking. Examination revealed marked truncal and proximal muscle weakness with preserved distal strength. Serum potassium was 2.7 mmol/L. His symptoms fully resolved following intravenous potassium replacement.

HPP is often underdiagnosed, and diagnostic delays can leave patients vulnerable to avoidable complications, including recurrent episodes and potentially life-threatening arrhythmias. This case highlights the importance of considering HPP in young patients presenting with acute muscle weakness and hypokalaemia.

## Introduction

Hypokalaemic periodic paralysis (HPP) is a rare neuromuscular disorder characterized by recurrent episodes of acute, transient muscle weakness with hypokalaemia (serum potassium <3.5 mmol/L) [1].

These episodes typically present with flaccid proximal muscle weakness and reduced deep tendon reflexes. Familial HPP follows an autosomal dominant inheritance pattern and is most commonly associated with loss-of-function mutations in the CACNA1S and SCN4A genes, which encode calcium and sodium skeletal muscle channels, respectively [2]. These mutations result in dysfunctional ion channels that inhibit muscle contractility.

The condition typically presents in childhood or adolescence, and there is often a positive family history [1]. A clinical diagnosis is usually made after multiple paralysis episodes with concurrent hypokalaemia and is confirmed with genetic testing [3]. Common triggers include high-carbohydrate meals, intense exercise, stress, and certain medications. Delayed treatment can lead to serious, life-threatening complications such as respiratory failure and cardiac arrhythmias; however, the condition often goes underdiagnosed due to its variable clinical presentation and limited awareness [3].

Acute management of HPP depends on symptom severity and duration. Some patients may require oral or intravenous potassium replacement, while in some cases, symptoms may resolve spontaneously [1]. Long-term management includes lifestyle modifications to avoid known triggers, adherence to a potassium-rich diet, and the use of medications such as carbonic anhydrase inhibitors, beta blockers, or potassium-sparing diuretics to prevent further attacks. In secondary or acquired cases of HPP, treatment should be guided by etiology, with management focused on addressing the underlying cause. We present a report of a clinical presentation of primary HPP, which highlights the importance of early recognition and treatment.

This case was submitted as a poster presentation to the Emergency Physicians International Conference (EPIC) 2025.

## Case presentation

A 17-year-old male presented to the Emergency Department with acute-onset transient limb paralysis upon waking earlier that morning. He denied any loss of consciousness, paresthesia, vertigo, headache, or changes in speech or vision. Notably, he had eaten a carbohydrate-rich meal the night before. He had no past medical history and took no regular medications. Both of his younger brothers had experienced similar episodes of transient paralysis at the ages of 13 and 15, although these were never investigated.

His observations, including blood pressure, were stable. On examination, he had marked truncal and proximal muscle weakness (MRC power scale 1/5) with preserved distal strength (power 5/5). There were no other neurological findings, and a cardiovascular examination was unremarkable.

**Figure 1:**
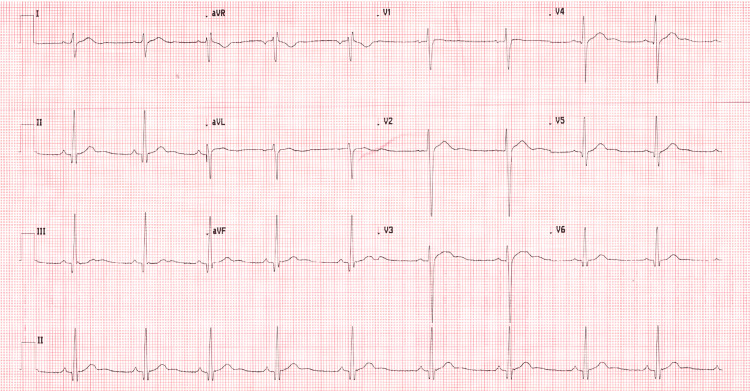
Admission ECG showing sinus bradycardia with U waves in leads II, aVF, V2, and V3 Prominent U waves indicate delayed ventricular repolarization and increased risk of arrhythmias [3].

Electrocardiogram showed sinus bradycardia (heart rate 56 bpm) with U waves in leads II, aVF, V2, and V3 (see Figure 1). Serum potassium was 2.7 mmol/L (normal serum potassium 3.5 to 5.5 mmol/L) [4], with normal magnesium and thyroid function. Renin-aldosterone ratio was normal, with normal cortisol and dexamethasone suppression test results. CT head and MRI head showed no acute intracranial abnormality. The patient received five intravenous potassium infusions (40 mmol each), resulting in complete resolution of leg weakness within 24 hours. He was discharged home 48 hours later. Approximately one month later, he re-presented with similar symptoms following episodes of diarrhea, with a potassium level of 2.9 mmol/L. His symptoms again resolved following intravenous potassium administration. Following this second presentation, the patient was offered further diagnostic evaluation, including genetic testing, which he declined. 

## Discussion

HPP is a rare disorder, with an estimated prevalence of approximately 1 in 100,000 [5]. It shows a male predominance and typically presents around the age of 14 years [6]. Like other skeletal muscle channelopathies, HPP is often underdiagnosed, with many patients receiving a diagnosis only after repeated episodes [7]. In this case, despite the classic features (a young male, identifiable triggers, positive family history, and rapid resolution with potassium replacement), the diagnosis was made only after two presentations, consistent with the pattern reported in the literature. Underdiagnosis is thought to arise from several factors, including variability in presentation and severity, long intervals between attacks, and limited clinical awareness [7].

When diagnosing HPP, it is essential first to exclude more common and potentially life-threatening causes of acute weakness. Neuroimaging was performed to rule out acute neurological presentations such as stroke, space-occupying lesions, or postictal paralysis [8]. Biochemical investigations helped exclude metabolic and endocrine disorders that can mimic HPP while also identifying secondary causes of hypokalaemic paralysis. For example, thyrotoxic periodic paralysis presents with transient weakness and hypokalaemia with hyperthyroidism [8,9]. Andersen-Tawil syndrome, another autosomal dominant channelopathy, is characterized by episodic weakness with cardiac arrhythmias and dysmorphic features [3]. HPP should also be differentiated from other causes of acute flaccid paralysis, such as Guillain-Barré syndrome, a demyelinating peripheral neuropathy that typically presents with progressive, ascending weakness, areflexia, and sensory impairment. In contrast, HPP results from impaired muscle contraction rather than a problem with nerve conduction; tendon reflexes are often reduced, while sensation remains intact.

Early recognition of HPP is crucial, as severe episodes can have significant and potentially life-threatening consequences. While rare, involvement of the respiratory muscles can lead to acute respiratory failure, and hypokalaemia can cause cardiac arrhythmias [6]. Furthermore, sudden onset of paralysis during activities such as swimming or driving poses additional safety risks. Prompt diagnosis allows clinicians to initiate early treatment and minimize complications [10].

Genetic testing plays an important role by confirming the diagnosis, classifying the specific HPP subtype, and guiding family screening, thereby informing long-term management strategies.

Management of HPP remains challenging, as most treatment strategies are based on case reports and anecdotal evidence rather than prospective studies, given the low prevalence of the condition. Adolescents may face additional challenges, including poor compliance with treatment and reduced engagement with follow-up and counseling, which place them at greater risk of recurrent episodes [11].

Given that this report details a single patient case, the findings may not be generalizable, particularly for patients with atypical presentations or late-onset disease. Additionally, the relatively short follow-up period, limited to one year after the second episode, restricts conclusions regarding long-term treatment efficacy and prognosis.

## Conclusions

This case demonstrates the importance of considering HPP in young patients presenting with acute weakness and hypokalaemia. Early recognition facilitates prompt treatment, reduces complications, and helps prevent recurrent episodes. Greater clinical awareness, patient education, and access to genetic testing are important to ensure accurate diagnosis and effective management of this condition.
